# The role of C-afferents in mediating neurogenic vasodilatation in plantar skin after acute sciatic nerve injury in rats

**DOI:** 10.1186/s12868-020-00564-6

**Published:** 2020-04-16

**Authors:** Tao Zhang, Jianhui Niu, Yaxian Wang, Junying Yan, Wen Hu, Daguo Mi

**Affiliations:** 1 Department of Radiology, The Third People’s Hospital of Nantong City and The Third Nantong Hospital Affiliated to Nantong University, Nantong, 226001 Jiangsu China; 2 Key Laboratory for Neuroregeneration of Ministry of Education and Co-innovation Center for Neuroregeneration of Jiangsu Province, Nantong University, Nantong, 226001 Jiangsu China; 3 School of Medicine, Nantong University, Nantong, 226001 Jiangsu China; 4 Department of Orthopedics, Nantong City Hospital of Traditional Chinese Medicine, Nantong, 226001 Jiangsu China; 5 Present Address: Department of Neurochemistry, Inge Grundke-Iqbal Research Floor, New York State Institute for Basic Research in Developmental Disabilities, Staten Island, NY 10314 USA

**Keywords:** Vasomotor control, Skin, C-fiber afferents, Nerve transection, Sciatic nerve deafferentation, Rats

## Abstract

**Background:**

Vasomotor regulation of dermal blood vessels, which are critical in the function of the skin in thermoregulatory control, involves both neural and non-neural mechanisms. Whereas the role of sympathetic nerves in regulating vasomotor activities is comprehensively studied and well recognized, that of sensory nerves is underappreciated. Studies in rodents have shown that severance of the sciatic nerve leads to vasodilatation in the foot, but whether sympathetic or sensory nerve fibers or both are responsible for the neurogenic vasodilatation remains unknown.

**Results:**

In adult Sprague–Dawley rats, vasodilatation after transection of the sciatic nerve gradually diminished to normal within 3–4 days. The neurotmesis-induced neurogenic vasodilatation was not detectable when the sciatic nerve was chronically deafferentated by selective resection of the dorsal root ganglia (DRGs) that supply the nerve. Specific activation of C-afferents by intra-neural injection of capsaicin resulted in neurogenic vasodilatation to a magnitude comparable to that by neurotmesis, and transection of the sciatic nerve pre-injected with capsaicin did not induce further vasodilatation.

**Conclusions:**

Our results collectively indicate that vasodilatation after traumatic nerve injury in rats is predominantly mediated by C-fiber afferents.

## Background

Vasomotor activity of blood vessels in the skin plays a pivotal role in thermoregulatory control of the body system. Blood vessels in the skin dilate or constrict in response to thermal challenges, facilitating the maintenance of thermal homeostasis of the body [1, 2]. For instance, dermal vasculature dilates in response to increase in whole body temperature, whereas it constricts as the body or ambient temperature drops to a certain level [1, 3]. It is known that both neural and non-neural (humoral) mechanisms are involved in regulation of vasomotion of the dermal vasculature; however, the neural control of dermal vasomotion can be dominant under certain circumstances [1]. The neural control of dermal vasomotion is likely mediated by nerve fibers that either innervate or travel alongside the blood vessels in the skin [4].

Resting skin blood flow is governed by dermal vascular tone which is orchestrated by the balance between vasoconstrictor neurotransmitters/neuromodulators released from sympathetic nerve fibers at the outer surface of the blood vessels and vasodilator substances released by the endothelium [5]. Sympathetic innervation is known to be responsible for reflex neural control of skin blood flow, which is regulated by both vasoconstrictor and vasodilator axons in sympathetic nerves in non-glabrous skin in humans [3]. However, the glabrous skin areas-such as palms, soles and lips where numerous arteriovenous anastomoses are present-are richly innervated by sympathetic vasoconstrictor but not vasodilator nerve fibers [1]. The mechanisms for how sympathetic vasoconstrictors act on skin blood flow have been unraveled by comprehensive studies, whereas those for sympathetic vasodilator nerve seem more complex and remain poorly understood [1, 3].

Vasomotor activities of dermal vasculature in response to local thermal challenges mainly involve neural control mechanisms, including both sympathetic and sensory nerve fibers [1, 6]. In previous studies, we found that both baseline blood perfusion and local thermal challenges-induced perfusion change in the skin were altered after peripheral nerve injury [7–9]. In rats, transection injury to the sciatic nerve or tibial nerve promptly resulted in a substantial increase in blood perfusion by several fold in ipsilateral plantar skin [8, 9], whereas chronically denervated plantar skin did not exhibit a significant difference in blood perfusion as compared to the contralateral normal side [9]. It remains elusive whether sympathetic nerve fibers or sensory ones or both are responsible for neurogenic vasodilatation following nerve injury. In the present study, we tested in rats the role of C-fiber afferents in nerve injury-induced vasodilatation by employing selective resection of the dorsal root ganglia (DRGs) that supply the sciatic nerve and intra-neural injection of capsaicin, the pungent ingredient of hot chili peppers that specifically activates C-afferent nerve fibers [10, 11].

## Results

### Sciatic nerve transection leads to acute vasodilatation in glabrous skin in the foot

**Fig. 1:**
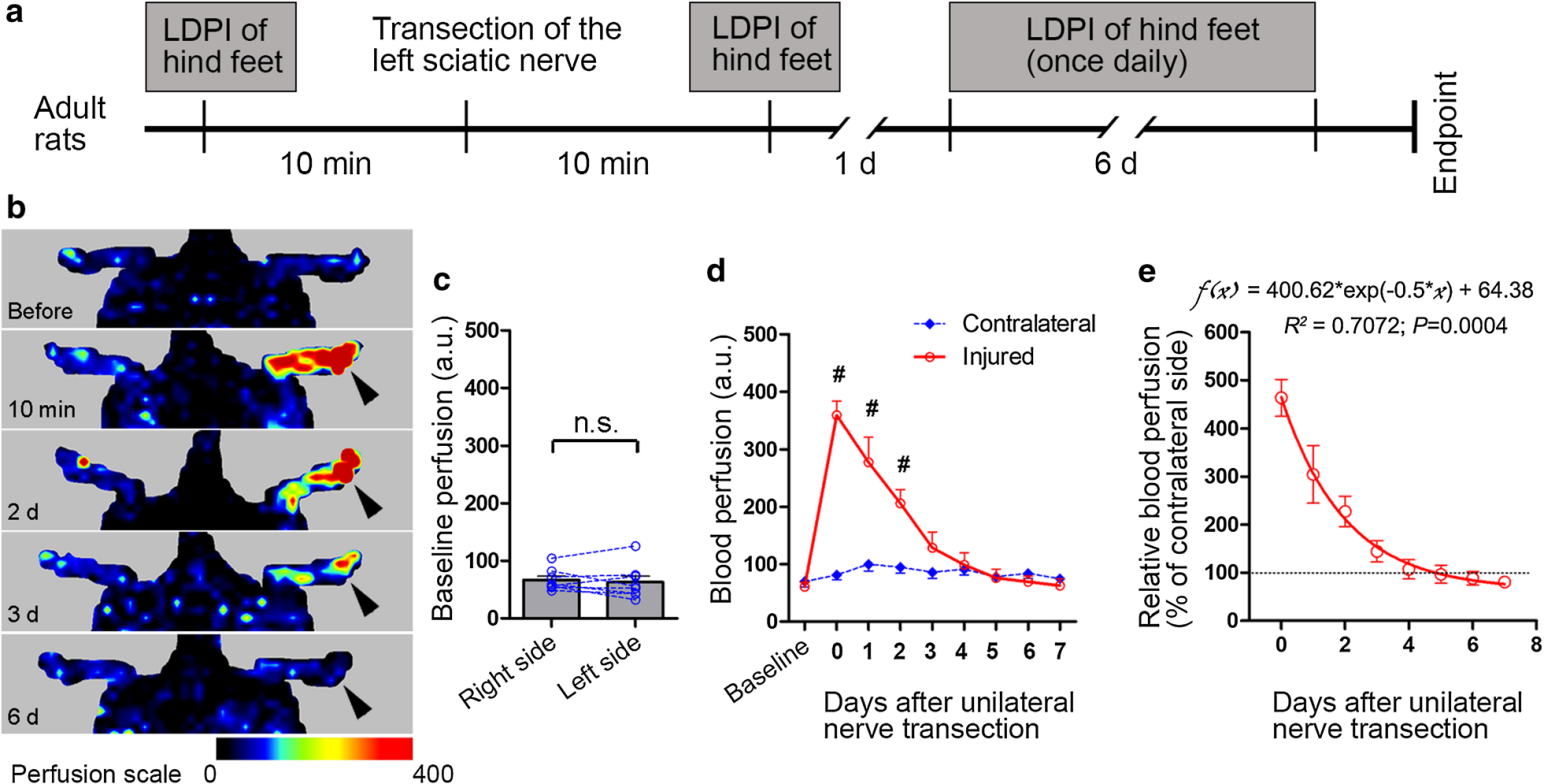
Sciatic nerve transection evokes prompt vasodilatation in the plantar aspect of the hind foot which rapidly decays in rats. **a** Schedule of experiments. The left sciatic nerve was transected at the mid-thigh level and laser Doppler perfusion imaging (LDPI) of the plantar aspect of both hind feet was performed before, 10 min after the nerve injury and then once daily for the following 6 days. **b** Representative LDPI images of the feet taken at indicated time. Color coding of the perfusion scale is shown at the bottom. **c** Bar chart with before-after lines showing the baseline perfusion in hind feet before injury. **d** Line chart demonstrating the change of blood perfusion in the hind feet of both sides from before to 7 days after transection of the left sciatic nerve. **e** One-phase decay curve fit of relative blood perfusion across time following nerve injury. Data are expressed as mean ± SEM (n = 8 rats) and analyzed with paired *t* test (**c**), repeated measures (mixed model) ANOVA followed by Bonferroni’s multiple comparison test (**d**) or non-linear regression and Spearman correlation (**e**). ^#^*P *< 0.001 compared to the contralateral side at the same time point. Arrowheads indicate the side with sciatic nerve injury. *n.s.* non-significant

To assess whether the two hind feet exhibit comparable levels of baseline perfusion, we first conducted LDPI analysis of the hind feet before sciatic nerve injury (Fig. 1a). We found that there was no statistically significant difference in the glabrous skin between the two hind feet in naive rats (Fig. 1a–c), suggesting that one hind foot can be used as an internal control to the other with regard to dermal perfusion. We then performed complete transection of the left sciatic nerve, and conducted LDPI analysis of the hind feet 10 min later and once daily until 7 days after nerve injury. We observed that transection of the rat sciatic nerve resulted in a prompt and robust increase in blood perfusion in the glabrous skin territory in the ipsilateral hind foot; however, the vasodilatation response to nerve injury was attenuated with time and almost disappeared at 3–4 days after nerve injury (Fig. 1b, d). The blood perfusion of the contralateral hind foot remained around the baseline level up to 7 days after nerve injury (Fig. 1d), indicating that the contralateral foot exhibited negligible vasodilatation after unilateral sciatic nerve injury and thus could serve as a reference. Therefore, we calculated the relative blood perfusion increase in the affected foot, i.e. percent of that on the contralateral side, against time after injury. We found that the one-phase decay model favorably fit the dynamic change in the level of vasodilatation, with a coefficient of determination of 0.7072 (Fig. 1e), suggesting a predictable change in the level of neurogenic vasodilatation after acute nerve injury in rats.

### Sciatic nerve deafferentation abolishes neurotmesis-induced vasodilatation

**Fig. 2:**
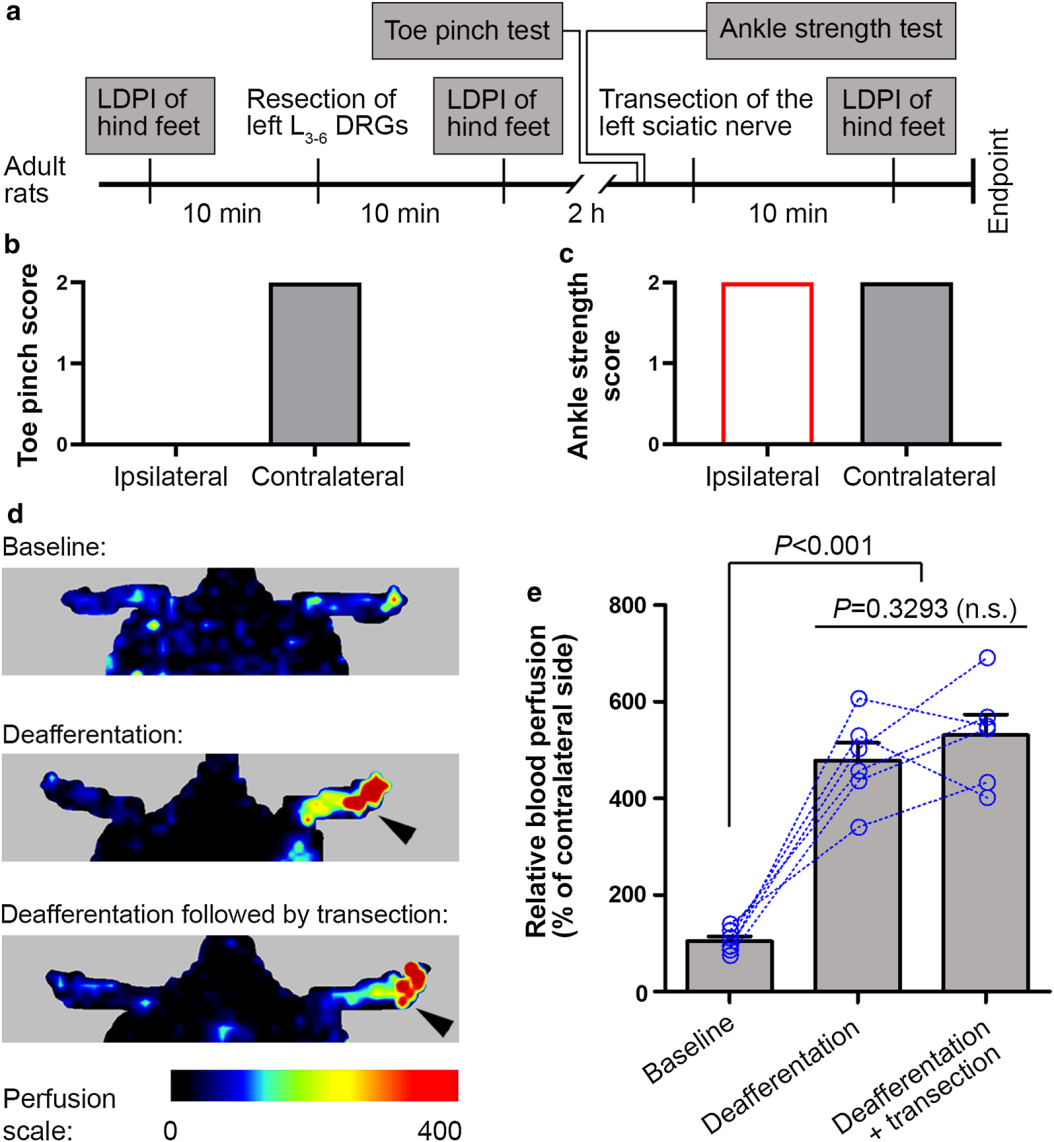
Deafferentation of the sciatic nerve results in prompt vasodilatation in the ipsilateral hind foot that does not further increase after transection of the nerve. **a** Experimental schedule. **b**, **c** Bar charts showing sensory and motor function 1.5 h after selective resection of L_3_–L_6_ dorsal root ganglia (DRGs) of the left side. The contralateral side served as an internal control. **d** Representative perfusion images of the hind feet before (baseline), 10 min after DRG resection (deafferentation), and after transection of the deafferentated sciatic nerve 2 h after DRG resection. Arrows indicate the side ipsilateral to surgery. **e** Bar chart with paired scattered dots showing the change of blood perfusion in the foot sequentially after DRG resection and nerve transection that followed. Data are expressed as mean ± SEM (n = 6 rats) and analyzed with repeated measures (mixed model) ANOVA followed by Bonferroni’s multiple comparison test (**e**). *n.s.* non-significant

To identify the role of C-fiber afferents in neurotmesis-induced vasodilatation, we first performed sciatic nerve deafferentation by resection of the left L_3_–L_6_ DRGs, which are known to supply the sciatic nerve [12, 13] and verified the successfulness of deafferentation (Fig. 2a). Assessed at 1.5 h after the surgery, when rats were fully recovered from anesthesia, toe pinch tests showed complete loss of sensation in the fifth toe on the ipsilateral side, all scored 0 without exception as compared to score 2 on the contralateral normal side (Fig. 2b). In addition, ankle strength test revealed no overt difference between the ipsilateral and contralateral hind feet; the strength was scored 2 on both hind feet in all rats with DRG resection (Fig. 2c). These data indicate complete deafferentation of the sciatic nerve, with the ventral roots left intact.

LDPI analysis showed that resection of L_3_–L_6_ DRGs resulted in a robust increase in blood perfusion in the foot 10 min after the surgery (Fig. 2d, e). Importantly, there was no additional increase in blood perfusion when the deafferentated sciatic nerve was further transected at the mid-thigh level at 2 h after DRG resection (Fig. 2d, e).

**Fig. 3:**
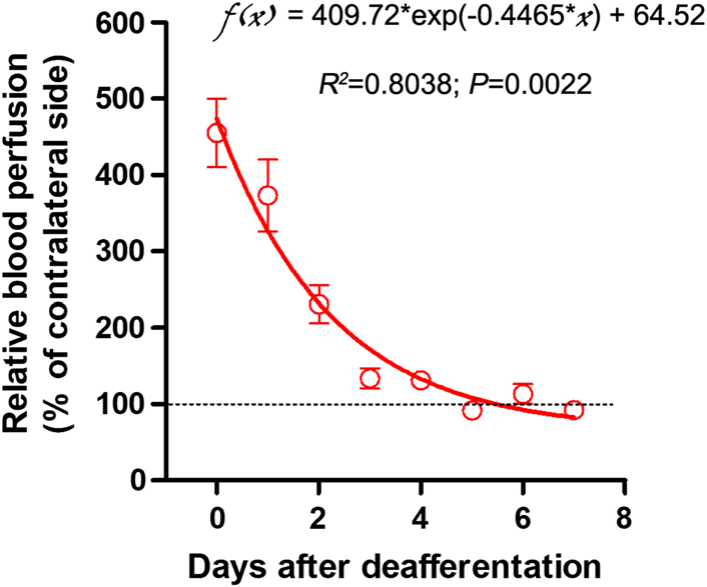
The increase in blood perfusion in the foot after sciatic nerve deafferentation rapidly drops back to normal. Data are expressed as mean ± SEM (n = 6 rats), analyzed with non-linear regression and Spearman correlation, and plotted with one-phase decay curve fit

To explore how the vasodilatation in the plantar glabrous skin after sciatic nerve deafferentation evolves over time, we performed daily LDPI analysis of the hind feet for 7 days in a separate cohort of rats after DRG resection. The dynamic change of vasodilatation showed a similar one-phase decay pattern with a coefficient of determination of 0.8038; the increase in blood perfusion after DRG resection gradually dropped back to normal within 3 days (Fig. 3).

**Fig. 4:**
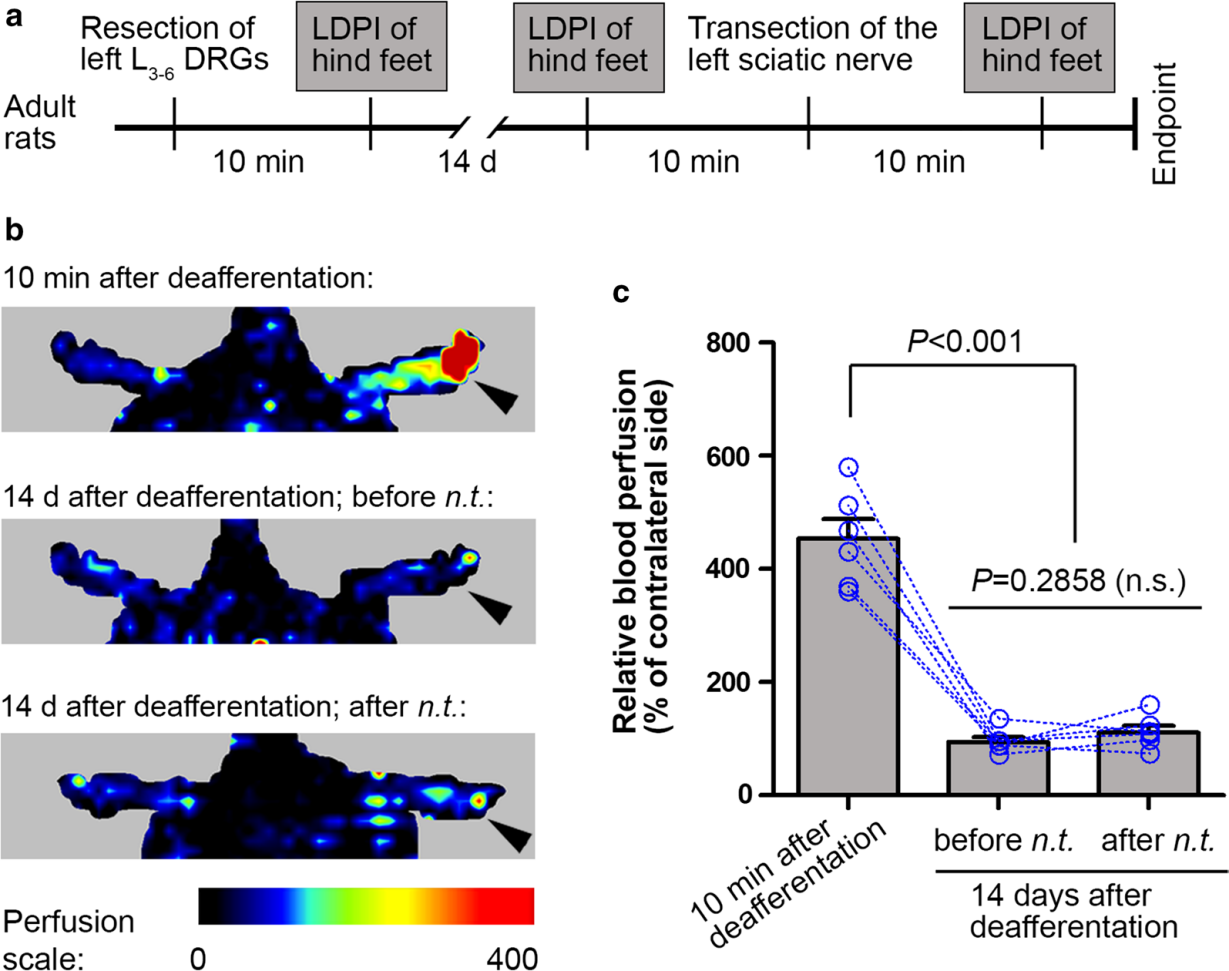
The foot did not exhibit vasodilatation 14 d post-deafferentation or after an additional surgery for transection of the ipsilateral sciatic nerve. **a** Experimental schedule. **b** Representative perfusion images of the hind feet promptly and 14 days after selective resection of L_3_–L_6_ dorsal root ganglia (DRGs) of the left side, and after transection of the left sciatic nerve. Arrows indicate the side ipsilateral to surgeries. **c** Bar chart with paired scattered dots showing the change of blood perfusion in the foot in the same rats. Data are expressed as mean ± SEM (n = 6 rats) and analyzed with repeated measures (mixed model) ANOVA followed by Bonferroni’s multiple comparison test. *n.s.* non-significant

We next tested whether there would be an increase in blood perfusion induced by transection of the deafferentated sciatic nerve at a stage when there was no detectable vasodilatation associated with DRG resection in the same cohort of rats for the LDPI assessment of dynamic change described above (Fig. 4a). Fourteen days after deafferentation surgery, the hind foot did not exhibit increase in blood perfusion as compared to the contralateral normal side (Fig. 4b, c). Intriguingly, vasodilatation was not evident in the foot after transection of the sciatic nerve which had been deafferentated for 14 days (Fig. 4b, c). This data, together with that observed acutely after DRG resection, suggests a dominant role of afferent nerve fibers in neurotmesis-induced vasodilatation.

### Transection of the nerve pre-injected with capsaicin does not induce additional vasodilatation

**Fig. 5:**
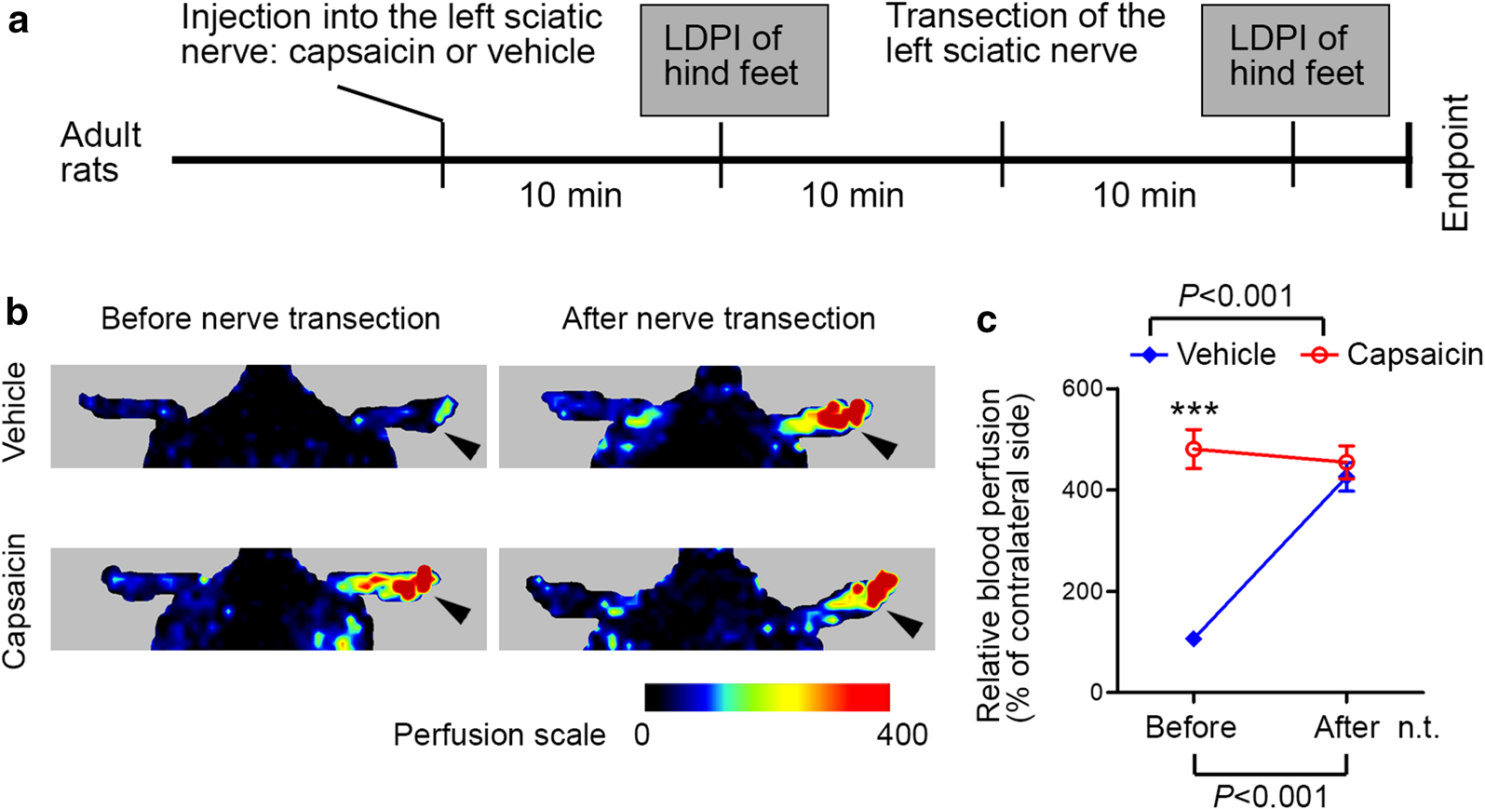
Injection of C-afferent irritator capsaicin into the sciatic nerve results in prompt vasodilatation in the foot that does not further increase in response to transection of the nerve. **a** Experimental schedule. **b** Representative perfusion images before and after transection of the left sciatic nerve. **c** Before-after line chart showing the change of blood perfusion in the foot. Data are expressed as mean ± SEM (n = 6 rats) and analyzed with repeated measures (mixed model) ANOVA followed by Bonferroni’s post hoc test. *P *< 0.001 for interaction between the group factor and the time factor, and for difference between capsaicin and vehicle before nerve transection (n.t.)

Capsaicin, the pungent ingredient of hot chili peppers and a vanilloid receptor subtype 1 agonist, is known to cause vasodilatation via specific activation of C-fiber afferents [14–16]. We took this advantage by injection of capsaicin in the rat sciatic nerve to specifically activate C-fiber afferents, and evaluated whether this prior activation of C-afferents would alter neurogenic vasodilatation after nerve transection. In contrast to injection of vehicle, a control group which did not show overt vasodilatation by itself, injection of capsaicin in the sciatic nerve induced a robust increase in blood perfusion in the hind foot which was comparable to that by transection of the nerve (Fig. 5a–c). However, transection of the sciatic nerve with prior injection of capsaicin did not induce further increase in blood perfusion in the foot (Fig. 5a–c). These results further confirm that C-afferents are the predominant mediator of neurogenic vasodilation after peripheral nerve injury.

## Discussion

Neural control of skin blood flow is thought to be attributable mainly to sympathetic nerves-involving sympathetic vasoconstrictor nerve fibers in most cases and sympathetic vasodilator nerve fibers in active dilation of vasculature in non-glabrous skin [1, 17]. In vertebrates, the regulating peripheral nervous system and the nourishing/regulating vascular system usually travel alongside each other and follow almost the same pattern of branching and distribution in the skin [4]. Whereas sympathetic innervation of cutaneous blood vessels and its control over vasomotor activity have been extensive studied and well recognized [5, 18–20], the role of sensory nerve fibers in neural control of vasomotion remains underappreciated to date [1, 6, 20]. In adult mammals, arteries and veins have been shown to be heavily innervated by sensory axons that contain calcitonin gene-related peptide (CGRP), substance P (SP) and/or neurokinin [21–23], in addition to innervation by dense network of sympathetic nerve fibers [24–26]. Intriguingly, a recent study has demonstrated that the arterial differentiation and blood vessel branching in the skin are intrinsically patterned by sensory nerves during development [4]. These studies suggest that sensory nerves may play a more important role in regulating cutaneous vasomotor activity than expected. Recent studies in humans also suggest that C-afferents play a role in mediating neurogenic vasodilation that is impaired in diabetes patients and in aged human skin [27–29]. In the present study we found, by using nerve deafferentation and C-afferent-specific stimulation models, that C-fiber afferents are predominantly responsible for neurogenic vasodilatation following traumatic nerve injury in the glabrous skin. These findings, together with our previous study which showed positive correlation between baseline perfusion in autonomous skin region and sensory recovery after peripheral nerve repair [7], suggest a pivotal role of afferent C-fibers in the regulation of blood flow in the skin, especially the glabrous regions of the skin.

Possible involvement of cutaneous polymodal nociceptor fibers (C-afferents) in mediating local dermal vasodilatation has been suggested by previous studies, the earliest of which was over one century ago [30–32]. Blumberg and Wallin applied painful electrical stimulation to the cutaneous fascicles of the superficial peroneal nerve at the ankle in healthy adults, and found that the intraneural stimulation induced marked vasodilatation in the dorsum of the foot; pre-blockade of the nerve proximal to the stimulation site with bupivacaine abolished the vasodilatation effect at the same stimulation strength [32]. It is worthy of noting that blockade of sensory fibers with regional anesthetics is helpful to study the sensory control of peripheral tissue and organs, but when employed in exploring the role of sensory nerve in vasomotor regulation, the data should be interpreted with caution. This is because sensory nerve blockade itself can result in marked vasodilatation in the skin innervated by the nerve, as shown by early studies in humans [33–35]. Dermal vasodilatation was also detectable 10–15 min after blockade of the femoral and sciatic nerves in dogs [36]. In this regard, the models of traumatic nerve injury and deafferentation offers a unique opportunity to study the role of sensory nerve fibers in vasomotor control.

Based on the known anatomical traits of nerves in the rat hind limb, including the location of neuronal somata that project sympathetic and afferent axons into the nerves and the organization of the lumbar sympathetic nervous system [37], we performed deafferentation of the left sciatic nerve to ensure depletion of C-afferents [13]. Deafferentation itself induced overt vasodilatation in the foot, and transection of the acutely deafferentated sciatic nerve did not further increase blood perfusion. It is of note that sympathetic nerve fibers, which presumably remained intact in the nerve deafferentation surgery, were disrupted by nerve transection. To exclude the possibility that the nerve deafferentation-induced vasodilatation would have reached the maximum capacity that might have masked any further effect by the additional nerve injury, we performed nerve transection 2 weeks after nerve deafferentation, when deafferentation-induced vasodilatation was no longer evident. The absence of vasodilatation after transection of the sciatic nerve that had been deafferentated for 2 weeks indicate that the vasodilatation induced by loss of sympathetic tone in glabrous skin in nerve injury in the rat, if any, may be limited or dependent on intact C-afferent innervation. Actually, it has been suggested from previous studies that sympathetic nerve can interact with sensory nerves [38–41]. Using a surgical sympathectomy model in adult rats, Ren et al. showed that the presence of sympathetic efferents is required for capsaicin-induced sensitization of C-afferents to mechanical stimuli and this modulation is α1-adrenergic receptors-dependent [41], in line with other studies which showed that sympathectomy alleviates mechanical allodynia in rats [38–40].

C-afferents are known to release vasoactive neuropeptides such as CGRP and SP, which are known to cause vasodilatation [42, 43]. In the present study specific activation of the C-afferents in the sciatic nerve with capsaicin induced neurogenic vasodilatation to a magnitude comparable to that by transection of the nerve, and transection of the sciatic nerve pre-injected with capsaicin did not induce further vasodilatation. These data, along with those observed after DRG resection, further indicate a dominant role of C-afferents in neurogenic vasodilatation induced by nerve injury.

**Fig. 6:**
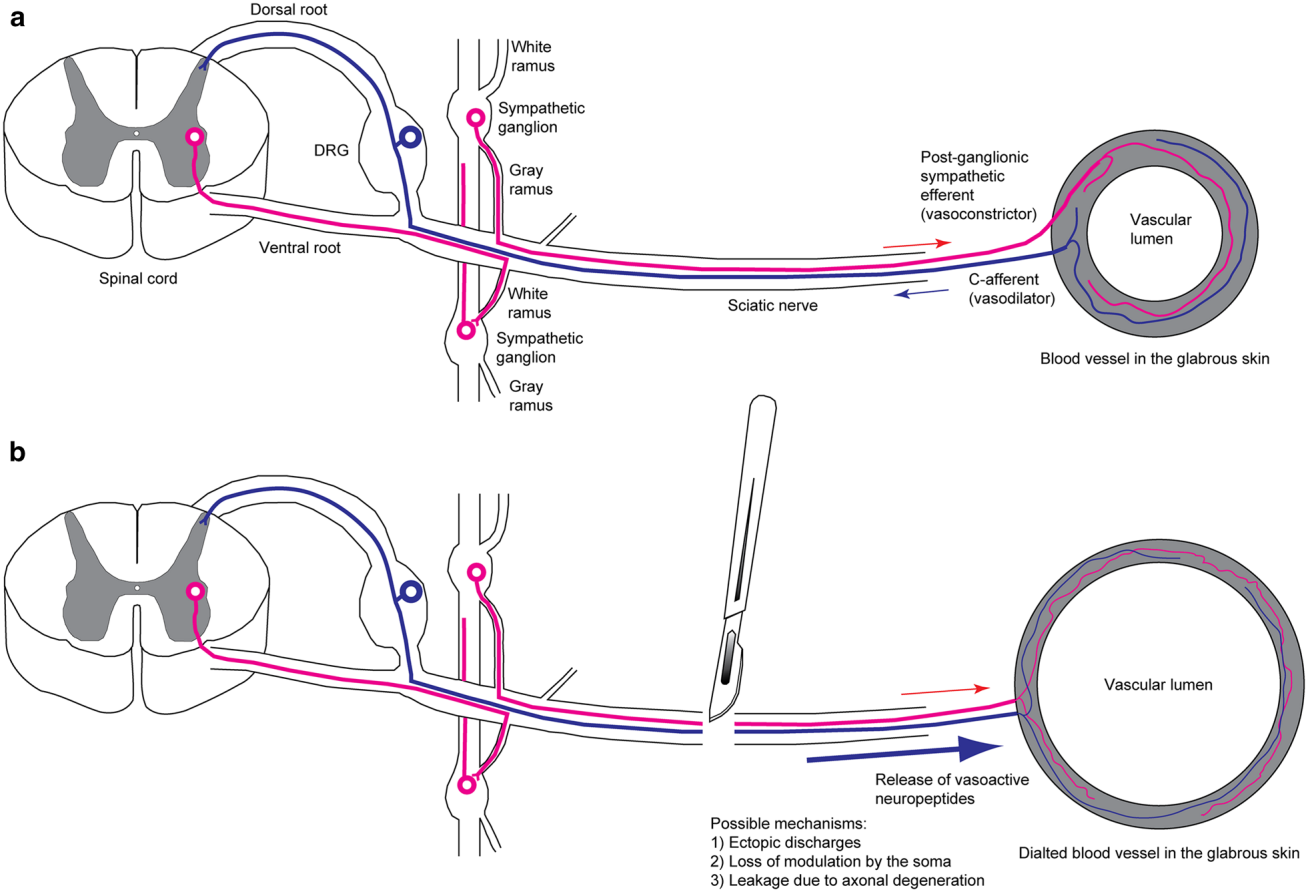
Proposed mechanisms of neurotmesis-induced vasodilatation. **a** Physiological condition. Neurogenic vasomotion is balanced by orthodromic vasoconstrictor activity of sympathetic axons and antidromic vasodilator activity of C-afferents. C-afferents may modulate sympathetic vasoconstrictor neurons via circuit in the spinal cord. **b** Neurotmesis-induced vasodilatation is predominantly mediated by C-afferents. Transection of the nerve trunk leaves the axonal distal segment of the C-afferent and the distal post-ganglionic sympathetic axon isolated from their soma, and may evoke antidromic and orthodromic discharges in C-afferents and post-ganglionic sympathetic axons in the distal nerve segment, respectively. Possible uncontrolled release of vasoactive neuropeptides from the C-afferents in the distal nerve segment may result from ectopic discharges, lack of modulation by the soma, and leakage during axonal degeneration

Cutaneous vasomotion in physiological condition appears to be governed in a balanced fashion by both sympathetic constrictor and C-afferent vasodilator, and possibly also by reflex between the two parties at the central end (Fig. 6a). Whereas the precise mechanism by which nerve transection evokes vasodilatation remains to be investigated, there are several plausible possibilities for it (Fig. 6b). First, transection of the nerve trunk leaves C-afferents and the postganglionic sympathetic axons in the nerve segment distal to the injury isolated from their soma and therefore, terminal release of vasoactive neuropeptides is no longer modulated by the soma and reflective interaction between sympathetic constrictor and C-afferent vasodilator is missing. Second, neurotmesis may evoke local biochemical change that can drive antidromic and orthodromic ectopic discharges in C-afferents and postganglionic sympathetic axons in the distal nerve segment, respectively. Finally, degeneration of the distal axonal segment may result in leakage of vasoactive neuropeptides from the isolated C-afferent fibers.

In the present study, the refinement and reduction of research animals were enforced by using minimal numbers of rats for each experiment, involving experienced and proficient experimenters in rodent surgery, and employing blind approach in data collection. A limitation of the present study is that the vascular physiology of rats may be different to some extent from that in humans, as indicated in our previous studies [7, 9]. However, as rodents are the most widely used models in studying neuro-vascular control mechanisms, the findings in the present study have implications in understanding the pathophysiological sequela of peripheral nerve injuries in humans and the development of novel assessment of nerve regeneration in experimental settings [44].

## Conclusions

In the present study we found that transection of the sciatic nerve in rats lead to neurogenic vasodilatation in glabrous skin in the foot which gradually diminished to normal within 3 to 4 days, that the neurogenic vasodilatation was not detectable if the nerve was chronically deafferentated, and that there is no further vasodilatation induced by nerve transection if the C-afferents are already activated. These results collectively indicate that vasodilatation after traumatic nerve injury in rats is predominantly mediated by C-fiber afferents.

## Methods

### Animals

Thirty-two adult female Sprague–Dawley non-breeder rats, 4–6 months of age and 298.5 ± 34.7 g in body weight, were used in the present study. The rats were specific pathogen free, originally from Charles River Laboratories (Wilmington, MA) and bred in Laboratory Animal Research Center at Nantong University. The animals were housed, 2 per cage in polycarbonate cages with corn cob beddings, in a 12-h light/dark schedule with ad libitum access to food and water in a barrier unit. All animal procedures, including surgeries and assessment, were performed at in the light phase. At the endpoint of each experiment, rats were euthanized by asphyxiation with carbon dioxide for 5 min at a flow rate of 2 L/min in a 10-L acrylic glass chamber, followed by pneumothorax as a secondary procedure. The verification of death and procedure for pneumothorax were performed as follows: the complete unconsciousness, absence of heartbeat and breath, and no response to toe pinpricking were verified in each animal; and then a 14-gauge injection needle was used to sequentially penetrate into each of bilateral thoracic cavities.

All animal procedures were carried out under the approval of the Ethics Committee for Laboratory Animals of Nantong University, China (approval No. 20130410-006) and in accordance with US National Institutes of Health Guide for the Care and Use of Laboratory Animals published by the US National Academy of Sciences. The ARRIVE guidelines/methodology originally published in PLoS Biology, June 2010 [45] were adhered to in reporting the in vivo experiments in the present study.

### LDPI analysis

Blood perfusion of the hind feet was measured by laser Doppler perfusion imaging (LDPI) using the PeriScan PIM 3 system (Perimed AB, Järfälla, Sweden) as previously described [8, 9]. Briefly, rats were allowed to be acclimated to the test room, 23 °C and 60% humidity, for at least 30 min before LDPI analysis. Rats were anesthetized with 3% sodium pentobarbital solution (30 mg/kg body weight), and placed in a prone position on a green soft pad, leaving the plantar aspect of both hind feet exposed to the laser beam overhead. The plantar side of the hind feet was then scanned twice with the repeated scan mode at a distance of ~ 18 cm. The arbitrary perfusion units measured from the two scanned images for each foot were averaged. In some cases a relative perfusion level of the foot on the affected side was expressed as percent of the contralateral normal side. LDPI analysis was performed by experimenters who were blind to experimental design and the identity of treatment groups, so were ankle strength test and toe pinch test described below.

### Nerve transection injury

The first cohort of naive rats (n = 8) was subjected to transection of the sciatic nerve. Rats were anesthetized with intraperitoneal injection of pentobarbital solution as described above, and the hair on the lateral thigh shaved. The plantar aspect of hind feet was subjected to LDPI analysis. Then an incision was made along the axis of the femoral shaft in the left lateral thigh, and the left sciatic nerve was exposed under aseptic condition and transversely lacerated at the mid-thigh level with sharp surgical scissors. The incision was closed in layers with 4/0 silk sutures. LDPI analysis of the hind feet was performed 10 min after the nerve transection injury. Rats were allowed to completely recover on a soft heating pad before returned to their home cages. Daily LDPI analysis of the hind feet was performed until 7 d after injury.

For other cohorts of animals described below, nerve transection injury was performed using the same surgical procedures as described for the first cohort.

Subcutaneous injection of ketoprofen (Zoetis Inc., Kalamazoo, MI, USA), 5 mg/kg body weight, was given in rats once daily for 3 days, starting immediately after the completion of surgery to relief post-operative pain. This also applied to any surgery described hereafter. For rats surviving for no more than 1 day after surgery, only one dose of ketoprofen was injected.

### Surgery for sciatic nerve deafferentation

Sciatic nerve deafferentation was performed by selective resection of the left L_3_–L_6_ DRGs in two cohorts of rats as previously described [12, 13]. Briefly, after rats were anesthetized with intraperitoneal injection of pentobarbital solution and baseline blood perfusion in the hind feet assessed with LDPI, the lumbar vertebrae were exposed via a dorsal midline incision and left hemilaminectomy was performed using a motorized mini drill to clearly expose the left L_3_–L_6_ DRGs. The DRGs were carefully isolated from the ventral roots and completely excised, with the ventral roots left intact. The incision was then closed in layers, and the hind feet were subjected to LDPI analysis as described above at 10 min after surgery. Rats were allowed to completely recover on a soft heating pad and subjected to toe pinch test before returned to their home cages. For relief of post-operative pain, kitoprofen was given as described above.

For one cohort of rats with sciatic nerve deafferentation (n = 6), the left sciatic nerve was transected at the mid-thigh level 2 h after DRG resection under anesthesia and LDPI analysis of the hind feet was performed 10 min later.

For the other cohort of rats with sciatic nerve deafferentation (n = 6), LDPI analysis of the hind feet was performed daily for 7 days and then at 14 days after DRG resection. Fourteen days after the deafferentation surgery, the left sciatic nerve was transected at the mid-thigh level and LDPI analysis of the hind feet was performed 10 min later.

### Ankle strength test

Ankle strength test was performed at ~ 1.5 h after DRG resection to assess the motor nerve function. Briefly, the awake rat was gently held by the base of the tail and its forelimbs were allowed to grasp the steel wires of the cage cover. The strength of the ankle was assessed by putting an index finger on the plantar aspect of the hind foot of the rat when it was trying to escape and pushed its hind feet backwards. Hind feet on both sides were assessed and a three-tier scoring paradigm was used: 0-paralyzed and no perceivable pushing back, 1-decreased strength as compared to normal, 2-strong pushing back with the strength indistinguishable from that of the contralateral normal foot.

### Toe pinch test

Toe pinch test was performed immediately after ankle strength test to validate deafferentation of the sciatic nerve, using the protocol described previously [46, 47] but with modification. Briefly, the awake rat was gently hand-held and the volar aspect of the second to fifth toes was gently pinched with a pair of eye dressing forceps. The pain perception response to pinch was scored based on the extent of hind limb withdrawal using a three-tier scale: 0-no response, 1-decreased response compared to normal, 2-strong and prompt withdrawal of the hind limb which is indistinguishable from the response of the counterpart on the contralateral normal side. The assessment was repeated three times and the highest score was selected to represent the response level. The naive toes on the contralateral side were also assessed as a reference.

### Intraneural injection of capsaicin

To further study the involvement of C-fiber afferents in neurotmesis-induced vasodilatation, rats was subjected to intra-neural injection of capsaicin to specifically irritate C-fiber afferents which has been shown to result in vasodilatation [48]. Rats of the fourth cohort were randomly divided into two groups: capsaicin and vehicle control (n = 6 rats each). The rats were first sorted by body weight, and by the date of birth in that case of the same body weight, and then randomized using the RAND function in Microsoft Excel and a pre-designated rule of odd/even group allocation.

After rats were anesthetized as described above, the left sciatic nerve was exposed and 2 µl of 0.5% capsaicin (Sigma, St. Louis, Missouri, USA) in sterile saline containing 10% ethanol and 10% Tween-80. For the vehicle control, rats received intraneural injection of the same volume of vehicle only. LDPI analysis of the hind feet was performed 10 min after injection. Two hours later, the left sciatic nerve was transected at the mid-thigh level and LDPI analysis of the hind feet was performed 10 min later to test whether the blood perfusion would further increase after nerve transection. The order of entry of rats in surgery was randomized using Microsoft Excel. For relief of post-operative pain, kitoprofen was given as described above.

### Statistical analysis

All quantitative data were analyzed with GraphPad Prism 5.0 software package. Sample size was determined based on our previous studies [8, 9], and no sample size estimation or power analysis was performed. Data were analyzed with paired *t* test, repeated measures analysis of variance (ANOVA) followed by Bonferroni’s post hoc test, or non-linear regression and Spearman correlation, where appropriate. For non-linear regression, the one-phase decay model was used for best fit of the data. *P *< 0.05 was considered statistically significant.

## Data Availability

All data are published in this article and materials can be obtained from the corresponding author upon reasonable request.

## Abbreviations

DRG: Dorsal root ganglia
LDPI: Laser Doppler perfusion imaging
ANOVA: Analysis of variance
CGRP: Calcitonin gene-related peptide
SP: Substance P

## References

[1] Charkoudian N (2003). Skin blood flow in adult human thermoregulation: how it works, when it does not, and why. Mayo Clin Proc.

[2] Kellogg DL (2006). In vivo mechanisms of cutaneous vasodilation and vasoconstriction in humans during thermoregulatory challenges. J Appl Physiol.

[3] Charkoudian N (2010). Mechanisms and modifiers of reflex induced cutaneous vasodilation and vasoconstriction in humans. J Appl Physiol.

[4] Mukouyama YS, Shin D, Britsch S, Taniguchi M, Anderson DJ (2002). Sensory nerves determine the pattern of arterial differentiation and blood vessel branching in the skin. Cell.

[5] Burnstock G, Ralevic V (2014). Purinergic signaling and blood vessels in health and disease. Pharmacol Rev.

[6] Hodges GJ, Traeger JA, Tang T, Kosiba WA, Zhao K, Johnson JM (2007). Role of sensory nerves in the cutaneous vasoconstrictor response to local cooling in humans. Am J Physiol Heart Circ Physiol.

[7] Deng A, Liu D, Gu C, Gu X, Gu J, Hu W (2016). Active skin perfusion and thermoregulatory response in the hand following nerve injury and repair in human upper extremities. Brain Res.

[8] Hu W, Liu D, Zhang Y, Shen Z, Gu T, Gu X, Gu J (2013). Neurological function following intra-neural injection of fluorescent neuronal tracers in rats. Neural Regener Res.

[9] Hu W, Yang M, Chang J, Shen Z, Gu T, Deng A, Gu X (2012). Laser doppler perfusion imaging of skin territory to reflect autonomic functional recovery following sciatic nerve autografting repair in rats. Microsurgery.

[10] Kenins P (1982). Responses of single nerve fibres to capsaicin applied to the skin. Neurosci Lett.

[11] Ilie MA, Caruntu C, Tampa M, Georgescu SR, Matei C, Negrei C, Ion RM, Constantin C, Neagu M, Boda D (2019). Capsaicin: physicochemical properties, cutaneous reactions and potential applications in painful and inflammatory conditions. Exp Therap Med.

[12] Hoke A, Redett R, Hameed H, Jari R, Zhou C, Li ZB, Griffin JW, Brushart TM (2006). Schwann cells express motor and sensory phenotypes that regulate axon regeneration. J Neurosci.

[13] Liu D, Zhang Y, Mi D, Gu J, Hu W (2013). A rat model of sciatic nerve deafferentation: anatomy, tracing and surgery. Chin J Hand Surg.

[14] Bonelli RM, Koltringer P (2000). Autonomic nervous function assessment using thermal reactivity of microcirculation. Clin Neurophysiol.

[15] Herbert MK, Holzer P (1994). Interleukin-1 beta enhances capsaicin-induced neurogenic vasodilatation in the rat skin. Br J Pharmacol.

[16] Lynn B, Ye W, Cotsell B (1992). The actions of capsaicin applied topically to the skin of the rat on C-fibre afferents, antidromic vasodilatation and substance P levels. Br J Pharmacol.

[17] Johnson JM, Minson CT, Kellogg DL (2014). Cutaneous vasodilator and vasoconstrictor mechanisms in temperature regulation. Compr Physiol.

[18] Greaney JL, Alexander LM, Kenney WL (2015). Sympathetic control of reflex cutaneous vasoconstriction in human aging. J Appl Physiol.

[19] Hodges GJ, Johnson JM (2009). Adrenergic control of the human cutaneous circulation. Appl Physiol Nutr Metab..

[20] Johnson JM, Kellogg DL (2010). Thermoregulatory and thermal control in the human cutaneous circulation. Front Biosci.

[21] Belai A, Milner P, Aberdeen J, Burnstock G (1996). Selective damage to sensorimotor perivascular nerves in the mesenteric vessels of diabetic rats. Diabetes.

[22] Buwalda J, Colnot DR, Bleys RL, Groen GJ, Thrasivoulou C, Cowen T (1997). Imaging and analysis of perivascular nerves in human mesenteric and coronary arteries: a comparison between epi-fluorescence and confocal microscopy. J Neurosci Methods.

[23] Holzer P (1998). Neurogenic vasodilatation and plasma leakage in the skin. Gen Pharmacol.

[24] Zou X, Lin Q, Willis WD (2002). The effects of sympathectomy on capsaicin-evoked fos expression of spinal dorsal horn GABAergic neurons. Brain Res.

[25] Renkin EM, Rosell S (1962). Independent sympathetic vasoconstrictor innervation of arterioles and precapillary sphincters. Acta Physiol Scand.

[26] Keatinge WR (1966). Electrical and mechanical response of arteries to stimulation of sympathetic nerves. J Physiol.

[27] Caselli A, Rich J, Hanane T, Uccioli L, Veves A (2003). Role of C-nociceptive fibers in the nerve axon reflex-related vasodilation in diabetes. Neurology.

[28] Marche P, Dubois S, Abraham P, Parot-Schinkel E, Gascoin L, Humeau-Heurtier A, Ducluzeau PH, Mahe G (2017). Neurovascular microcirculatory vasodilation mediated by C-fibers and Transient receptor potential vanilloid-type-1 channels (TRPV 1) is impaired in type 1 diabetes. Sci Rep.

[29] Munce TA, Kenney WL (2003). Age-specific skin blood flow responses to acute capsaicin. J Gerontol Ser A Biol Sci Med Sci.

[30] Bayliss WM (1901). On the origin from the spinal cord of the vaso-dilator fibres of the hind-limb, and on the nature of these fibres. J Physiol.

[31] Celander O, Folkow B (1953). The nature and the distribution of afferent fibres provided with the axon reflex arrangement. Acta Physiol Scand.

[32] Blumberg H, Wallin BG (1987). Direct evidence of neurally mediated vasodilatation in hairy skin of the human foot. J Physiol.

[33] Netten PM, Wollersheim H, Gielen MJ, Den Arend JA, Lutterman JA, Thien T (1995). The influence of ulnar nerve blockade on skin microvascular blood flow. Eur J Clin Invest.

[34] Saumet JL, Degoute CS, Saumet M, Abraham P (1992). The effect of nerve blockade on forearm and finger skin blood flow during body heating and cooling. Int J Microcirc Clin Exp.

[35] Campero M, Verdugo RJ, Ochoa JL (1993). Vasomotor innervation of the skin of the hand: a contribution to the study of human anatomy. J Anat.

[36] Gatson BJ, Garcia-Pereira FL, James M, Carrera-Justiz S, Lewis DD (2016). Use of a perfusion index to confirm the presence of sciatic nerve blockade in dogs. Vet Anaesth Analg.

[37] Baron R, Janig W, Kollmann W (1988). Sympathetic and afferent somata projecting in hindlimb nerves and the anatomical organization of the lumbar sympathetic nervous system of the rat. J Comp Neurol.

[38] Kim SH, Chung JM (1991). Sympathectomy alleviates mechanical allodynia in an experimental animal model for neuropathy in the rat. Neurosci Lett.

[39] Kim SH, Na HS, Sheen K, Chung JM (1993). Effects of sympathectomy on a rat model of peripheral neuropathy. Pain.

[40] Lin Q, Zou X, Fang L, Willis WD (2003). Sympathetic modulation of acute cutaneous flare induced by intradermal injection of capsaicin in anesthetized rats. J Neurophysiol.

[41] Ren Y, Zou X, Fang L, Lin Q (2005). Sympathetic modulation of activity in Adelta- and C-primary nociceptive afferents after intradermal injection of capsaicin in rats. J Neurophysiol.

[42] Benarroch EE (2011). CGRP: sensory neuropeptide with multiple neurologic implications. Neurology.

[43] Newby DE, Sciberras DG, Ferro CJ, Gertz BJ, Sommerville D, Majumdar A, Lowry RC, Webb DJ (1999). Substance P-induced vasodilatation is mediated by the neurokinin type 1 receptor but does not contribute to basal vascular tone in man. Br J Clin Pharmacol.

[44] Wang XS, Chen X, Gu TW, Wang YX, Mi DG, Hu W (2019). Axonotmesis-evoked plantar vasodilatation as a novel assessment of C-fiber afferent function after sciatic nerve injury in rats. Neural Regener Res.

[45] Kilkenny C, Browne WJ, Cuthill IC, Emerson M, Altman DG (2010). Improving bioscience research reporting: the ARRIVE guidelines for reporting animal research. PLoS Biol.

[46] Kovacic U, Zele T, Osredkar J, Sketelj J, Bajrovic FF (2004). Sex-related differences in the regeneration of sensory axons and recovery of nociception after peripheral nerve crush in the rat. Exp Neurol.

[47] Ma CH, Brenner GJ, Omura T, Samad OA, Costigan M, Inquimbert P, Niederkofler V, Salie R, Sun CC, Lin HY (2011). The BMP coreceptor RGMb promotes while the endogenous BMP antagonist noggin reduces neurite outgrowth and peripheral nerve regeneration by modulating BMP signaling. J Neurosci.

[48] Izumi H, Karita K (1990). The effects of capsaicin applied topically to inferior alveolar nerve on antidromic vasodilatation in cat gingiva. Neurosci Lett.

