# Correction to: The role of C-afferents in mediating neurogenic vasodilatation in plantar skin after acute sciatic nerve injury in rats

**DOI:** 10.1186/s12868-020-00568-2

**Published:** 2020-04-27

**Authors:** Tao Zhang, Jiahui Niu, Yaxian Wang, Junying Yan, Wen Hu, Daguo Mi

**Affiliations:** 1 Department of Radiology, The Third People’s Hospital of Nantong City and The Third Nantong Hospital Afliated to Nantong University, Nantong, 226001 Jiangsu China; 2 Key Laboratory for Neuroregeneration of Ministry of Education and Co-innovation Center for Neuroregeneration of Jiangsu Province, Nantong University, Nantong, 226001 Jiangsu China; 3 School of Medicine, Nantong University, Nantong, 226001 Jiangsu China; 4 Department of Orthopedics, Nantong City Hospital of Traditional Chinese Medicine, Nantong, 226001 Jiangsu China; 5 Present Address: Department of Neurochemistry, Inge Grundke-Iqbal Research Floor, New York State Institute for Basic Research in Developmental Disabilities, Staten Island, NY 10314 USA

## Correction to: BMC Neurosci (2020) 21:15 10.1186/s12868-020-00564-6

Following publication of the original article [1], the authors identified an error in the author name of Jiahui Niu.

The incorrect author name is: Jianhui Niu.

The correct author name is: Jiahui Niu.

The author group has been updated above and the original article [1] has been corrected.
